# Is There a Functional Role for the Knotted Topology in Protein UCH-L1?

**DOI:** 10.1021/acs.jcim.4c00880

**Published:** 2024-07-24

**Authors:** Sara G. F. Ferreira, Manoj K. Sriramoju, Shang-Te Danny Hsu, Patrícia F. N. Faísca, Miguel Machuqueiro

**Affiliations:** † BioISI - Instituto de Biossistemas e Ciências Integrativas, Departamento de Química e Bioquímica, Faculdade de Ciências, Universidade de Lisboa, 1749-016 Lisboa, Portugal; ‡ Institute of Biological Chemistry, Academia Sinica, Taipei 11529, Taiwan; § International Institute for Sustainability with Knotted Chiral Meta Matter (WPI-SKCM^2^), Hiroshima University, 1-3-1 Kagamiyama, Higashi-Hiroshima, Hiroshima 739-8526, Japan; ∥ BioISI - Instituto de Biossistemas e Ciências Integrativas, Departamento de Física, Faculdade de Ciências, Universidade de Lisboa, 1749-016 Lisboa, Portugal; ⊥ Institute of Biochemical Sciences, National Taiwan University, Taipei 11529, Taiwan

## Abstract

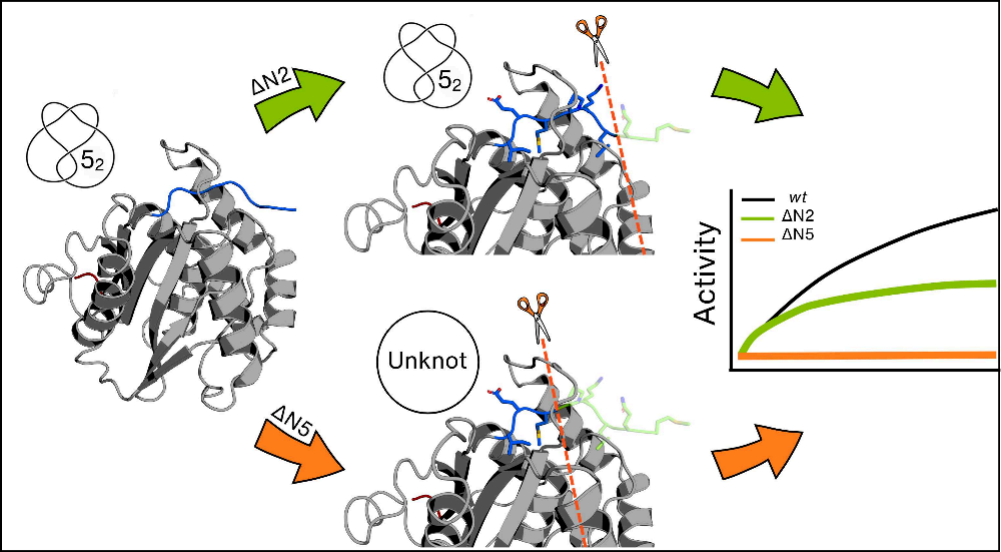

Knotted proteins are present in nature, but there is still an open issue regarding the existence of a universal role for these remarkable structures. To address this question, we used classical molecular dynamics (MD) simulations combined with *in vitro* experiments to investigate the role of the Gordian knot in the catalytic activity of UCH-L1. To create an unknotted form of UCH-L1, we modified its amino acid sequence by truncating several residues from its N-terminus. Remarkably, we find that deleting the first two N-terminal residues leads to a partial loss of enzyme activity with conservation of secondary structural content and knotted topological state. This happens because the integrity of the N-terminus is critical to ensure the correct alignment of the catalytic triad. However, the removal of five residues from the N-terminus, which significantly disrupts the native structure and the topological state, leads to a complete loss of enzymatic activity. Overall, our findings indicate that UCH-L1’s catalytic activity depends critically on the integrity of the N-terminus and the secondary structure content, with the latter being strongly coupled with the knotted topological state.

## Introduction

Knotted proteins are proteins whose native state embeds an open (i.e., physical) knot.^1^ The first reference to a knotted protein in the literature dates to 1977,^2^ but it was the development of a knot detection method in 2000,^3^ which allowed a systematic screening of the Protein Data Bank (PDB), that triggered the interest of the protein folding community in these intricate macromolecules. According to the latest survey, about 1% of the protein entries in the PDB correspond to knotted structures.^4,5^

Knots are classified according to the minimal number of crossings of the polypeptide chain on a planar projection of the knot. The most frequent knot type found in proteins is the 3_1_ (or trefoil) knot (with three crossings), followed by the 4_1_ (or figure-eight) knot (with four crossings), and the 5_2_ (or Gordian) knot (with five crossings). So far, only one protein was found in the PDB to embed the 6_1_ (or Stevedore’s) knot in its native structure,^6^ while a 7_1_ knot, the most topologically complex knot found to date, was reported for a protein structure predicted by AlphaFold^7^ and confirmed experimentally.^8^

The polypeptide chain of a knotted protein can be divided into three regions: the knotted core, which is the minimal chain segment that contains the knot, and two knot tails, which are the chain segments extending from each terminus to the knotted core. A deeply knotted protein should have at least one large knot tail since its knotted core is located far from the corresponding terminus. When the deletion of 20 or more residues from one of the termini is required to untie the chain, the knot classifies as deep; otherwise, it is considered shallow.^3,9^ Research on knotted proteins has been directed to solve two fundamental problems: 1) understanding their folding and knotting mechanisms (reviewed in^10,11^), and 2) identifying the knot’s functional role (reviewed in^12,13^).

Molecular simulations studies framed on models with different structural resolutions, ranging from lattice Monte Carlo simulations^14,15^ to full atomistic classical molecular dynamics,^16,17^ predict a common knotting mechanism based on a single threading event of the shortest knot tail through a loop formed by the remainder of the polypeptide chain. Experimental studies, focusing on kinetics and mechanisms, indicate that knotting is rate limiting, with some proteins (especially those with deep knots) populating transient intermediates, exhibiting parallel folding pathways, and slower folding rates.^18,19,19−22^ A systematic *in vitro ϕ*-value analysis (in which the knotted topology was preserved in the unfolded state), combined with restrained molecular dynamics have also reached a converging view of the smallest 3_1_ knotted MJ0366 protein being knotted in the transition state, which forms very late along the folding reaction coordinate.^23^ It is likely that knotting *in vivo* may be assisted by chaperonins,^24−27^ and computational studies also suggest that the ribosome may facilitate the knotting step.^28^

Despite their complex folding kinetics and mechanisms, it is known that knotted proteins are present in all kingdoms of life and tend to be conserved by nature. Thus, it is expected that knots may add an evolutionary advantage to their carriers by playing some functional role. The analysis of specific knotted proteins suggested that knots may enhance mechanical^29,30^ and kinetic stability,^21,31^ play a role against protein degradation,^30,32^ provide structural stability in transporter proteins,^33^ or even alter enzymatic activity.^34^ Recent simulation efforts predict that neither shallow nor deep knots should affect the thermodynamic equilibrium properties (such as the melting temperature).^35^ However, a consensus has not been achieved regarding the existence of a universal role for knots in proteins,^12^ and one should not put aside the possibility that they may not play any functional role at all.

**Figure 1:**
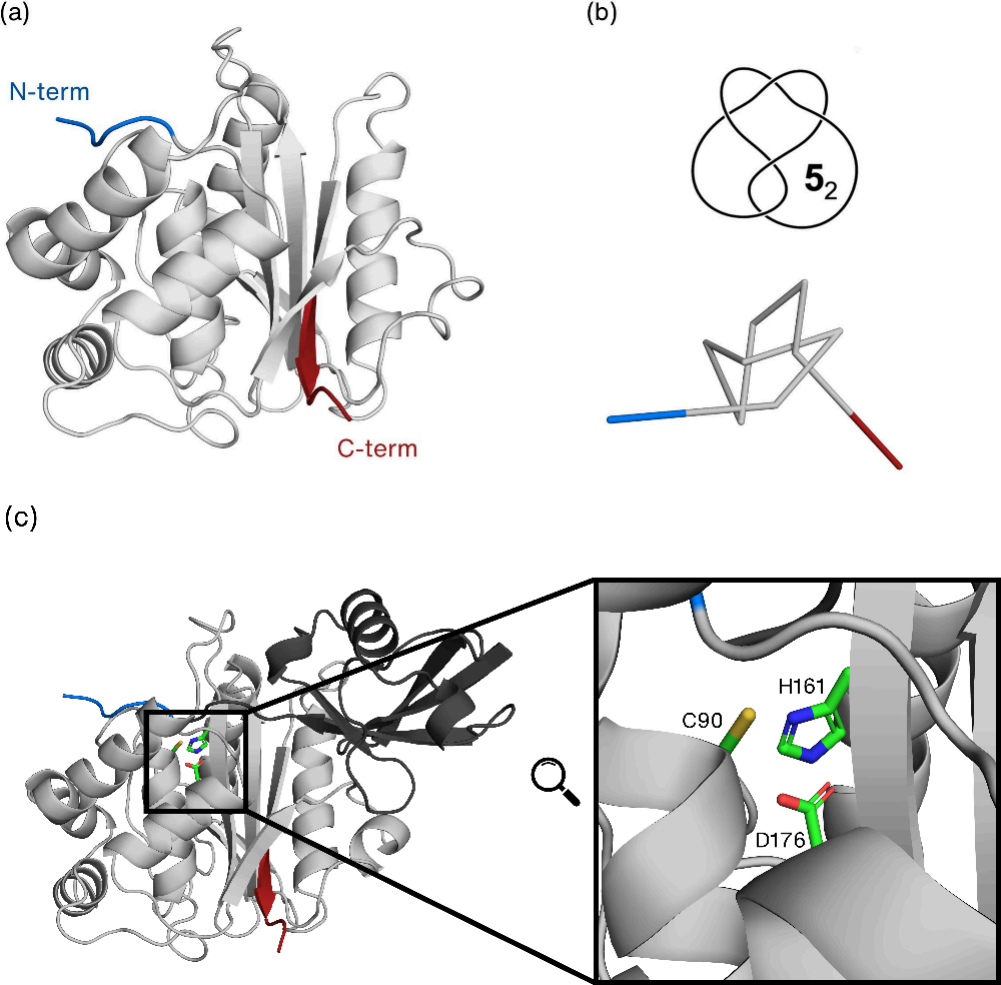
Protein UCH-L1. (a) Cartoon representation of the native structure of UCH-L1 (PDBid: 2ETL). The overall 3D structure results in two lobes of α-helices surrounding a tightly packed conserved hydrophobic core of β-strands. The knotted core (colored gray) extends from residue 6 to residue 217, and the knot tails are represented in blue (N-terminus) and red (C-terminus). (b) Diagrammatic representation of a reduced version of the backbone showing the five crossings of the 5_2_ knot. (c) The UCH-L1 complex with ubiquitin (cartoon shown in darker gray) where the catalytic triad residues (C90, H161, and D176) are highlighted and represented in green sticks.

With a few exceptions,^6,19,31,36^ most molecular simulation and experimental studies have been focused on proteins with trefoil knots.^16,17,23,26,27,35,37^ Therefore, here, we investigate the ubiquitin carboxy-terminal hydrolase L1 (UCH-L1), a single domain protein whose native structure embeds a 5_2_ (or Gordian) knot (Figure 1a-b). Besides its important role in the ubiquitin-proteasome system, UCH-L1 is highly expressed in several forms of cancer^38,39^ and is also one of the most abundant proteins in the brain, where it is estimated to make up 1 to 5% of the total neuronal protein.^40^ Its presence in Lewy bodies^41^ led to the hypothesis that it may be involved in the onset of Parkinson’s disease, Alzheimer’s disease, and other neurodegenerative diseases.^42,43^

Experimental studies *in vitro* show that UCH-L1 folds through parallel folding pathways and populates at least two metastable (unknotted) intermediate states, with knotting being the rate-limiting step.^20^ A consistent picture is observed via molecular simulations,^36^ while optical tweezers experiments reveal a more complex energy landscape with many on- and off-pathway intermediates being populated upon unfolding and refolding.^44^

The 5_2_ knot in UCH-L1 has been suggested to enhance mechanical stability and to protect it from proteasomal degradation,^30^ but the directionality of the mechanical pulling of the 5_2_ knot also makes a difference.^45^ Since the knotted core in UCH-L1 comprises both the substrate binding site and the catalytic site, one may hypothesize that the knot plays a role in catalysis by providing structural stability for UCH-L1’s catalytic triad. To the best of our knowledge, this question was not yet addressed experimentally, nor in the scope of molecular simulations. In the present study, we use classical molecular dynamics (MD) simulations, complemented by circular dichroism (CD) spectroscopy and activity measurements, to thoroughly investigate the role of the knot in the alignment and structural stabilization of the UCH-L1 catalytic triad when in a complex with ubiquitin.

## Model Systems

**Figure 2:**
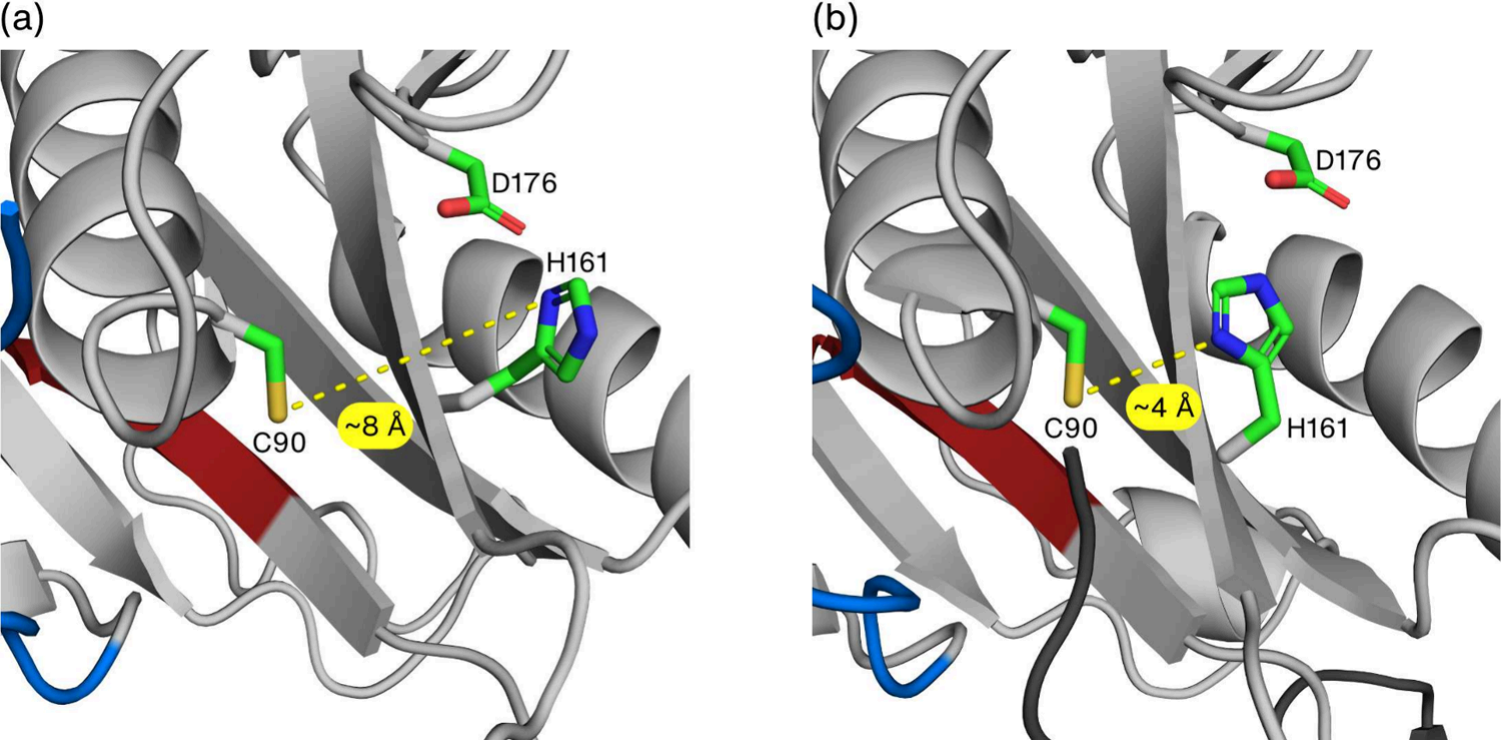
Distance between Cys90 (sulfur atom) and His161 (closest nitrogen atoms) in the apo (a) and holo or ubiquitin complex (b) states of UCH-L1. The knotted core and tails are represented in gray, blue (N-terminus), and red (C-terminus). The ubiquitin tail, inserted into the active site, is shown in a dark gray cartoon while the catalytic triad residues are highlighted as green sticks.

UCH-L1 is a single-domain protein with a chain length of 223 amino acids. Its native structure contains a 5_2_ knot, located between residues 6 and 217. The knot tails are short, being enough to remove five and six residues, from the N- or C-terminus, respectively, to untie the chain (Figure 1). From a biological point of view, UCH-L1 is a cysteine protease with a catalytic triad consisting of a cysteine (Cys90), a histidine (His161), and an aspartate (Asp176). In canonical cysteine proteases, the nucleophilicity of the catalytic cysteine is enhanced by the abstraction of the proton from the thiol group by a proximal histidine. To act as the general base, it is required for the imidazole group to be near the thiol (<4 Å). However, in UCH-L1 apo crystal structure (PDBid: 2ETL),^46^ the catalytic triad is misaligned for catalysis, with the His161 and Cys90 residues being ∼8 Å apart (Figure 2a). Interestingly, when UCH-L1 binds ubiquitin (PDBid: 3KW5),^47^ a conformational rearrangement occurs, bringing the residues of the catalytic triad into closer proximity (Figure 2b), and therefore promoting the enzymatic activity.

Within the UCH-L1 gene, several familial mutations were identified, such as I93M, E7A, and S18Y, which have been linked to neurodegenerative disorders.^40,43,48^ The E7A mutation exhibits a notable decrease in UCH-L1’s ability to bind to ubiquitin, which leads to a significant reduction in the protein’s deubiquitinating activity.^41^ The reduction in the ubiquitin-binding affinity and the overall UCH-L1 activity observed in E7A mutation is likely linked to conformational changes in the ubiquitin-binding pocket, near the catalytic cysteine, Cys90. Glu7 is conserved among all UCH protein family members and has been shown to be essential for the binding to ubiquitin.^40,46^

In the present work, we considered the apo state of UCH-L1 (PDBid: 2ETL^46^), and its holo state combined with ubiquitin, which is 76 residues long (PDBid: 3KW5(47)), and the final residue (G76), which was modified in the crystal, was rebuilt by using the Builder tool in PyMOL.^49^

Additionally, to investigate the role of the knotted topological state in the catalytic activity of UCH-L1, we prepared several truncated versions of UCH-L1, starting from both the apo and the holo systems. Our working hypothesis is that the deletion of residues from the protein termini may increase the probability of observing unknotting events in the course of an MD trajectory, allowing us to explore the relation between the catalytic activity and the topological state. Therefore, for each considered (apo and holo) system, a truncated variant is computationally generated by removing one amino acid at a time from the N-terminus (or from the C-terminus). This procedure generated 8 truncated variants for either terminus to ensure that both knot tails eventually become unthreaded. We named apo-*Δi* (and holo-*Δi*), the truncated variants obtained by removing *i* residues (with 1 ≤ *i* ≤ 8) from either the N-terminus or the C-terminus. It should be noted that the physical significance of the truncated variants generated computationally is limited to the ability of their *in vitro* engineered counterparts to fold into a well-defined native structure similar to that of the *wt*. One cannot exclude the possibility that in some cases the truncation will lead to a significant loss of secondary structure (such as that experimentally observed for ΔN11^30^), which will not be accessible within the timescale of the MD simulations. Since it is not feasible to experimentally investigate all the considered truncated variants, we focus the *in vitro* analysis on ΔN2 and ΔN5. An L3M mutation was also introduced in the ΔN2 protein to enable bacterial expression. To compare the computational results with the experimental ones, we also generated the ΔN2-L3M mutant. Both the E7A and ΔN2-L3M mutants were prepared using the mutagenesis tool of PyMOL.^49^

## Materials and Methods

### Molecular Dynamics Simulations

MD simulations were performed in all systems (apo, holo, and truncated versions) using the GROMACS 2021.2 software package^50^ and the GROMOS 54A7 force field.^51^ The initial configuration of the apo system contained 6500 water molecules, and 8 Na^+^ ions, which were added for charge neutrality. In the case of the holo system, 14000 water molecules, and 8 Na^+^ ions were added. For both systems, the protonation states of the different amino acids were estimated from p*K*_a_ calculations using the PypKa tool.^52^ We confirmed that no unusual protonations were detected at pH 7.0, even for histidines, which were all kept neutral. For the truncated systems, small adjustments were made to the number of water molecules and counterions to account for changes in the systems’ size and charge, upon truncation.

SPC^53^ was used to model the water molecules. Particle interactions were calculated within a cutoff radius of 1.4 nm for all interaction pairs, while the long-range electrostatic interactions were truncated (Lennard-Jones) or computed using the Particle-Mesh Ewald (PME) method (Coulombic).^54^ The PME calculations employed a grid spacing of 1.2 Å and a fourth-order interpolation scheme. To control and maintain the system at physiological temperature (310 K), we used the v-rescale thermostat,^55^ with a relaxation time of 0.1 ps. Pressure control was achieved using the Parrinelo–Rahman barostat^56^ with a semi-isotropic coupling at 1 bar. An isothermal compressibility value of 4.5 × 10^–5^ bar^–1^ and a relaxation time of 2.0 ps were employed for the pressure control. For all systems, a time step of 2 fs was used for the integration of the equations of motion, and the p-LINCS constraint algorithm was employed to constrain the bonds involving the solute atoms.^57^

To ensure a stable starting system, we performed two steps of energy minimization. Initially, 2000 steps of unconstrained steepest descent minimization were done, followed by ∼100 steps of LINCS-constrained minimization. After the minimization procedure, we initialized the temperature (NVT) and pressure (NPT) baths, which were equilibrated with short MD segments (100 ps). During this equilibration phase, the protein’s heavy atoms were harmonically restrained using a force constant of 1000 kJ mol^–1^ nm^–2^. Following this equilibration, MD runs regarding the apo systems were performed for 500 ns, while in the case of the ubiquitin-complex (holo) systems, the simulations were extended to 1 μs. To improve our sampling and ensure statistical rigor, five replicates of all systems were performed. Conformational snapshots were saved at regular intervals (100 ps) throughout the simulation trajectory. To ensure system equilibration, we discarded the initial 250 (500) ns of the apo (holo) system MD trajectories.

### Structural Properties and Binding Free Energy

To assess the structural stability of both UCH-L1 and ubiquitin, we calculated the positional Root Mean Square Deviation (RMSD) of the C_α_ atoms, the radius of gyration (*R*_g_), and the percentage of secondary structure (α-helices and β-strands content) using the DSSP algorithm.^58^

For each ubiquitin-complex system, the Solvent-Accessible Surface Area (SASA) was used to calculate the contact area between UCH-L1 and ubiquitin.^59,60^ This property allows assessing the structural stability of the protein–substrate complex.

To evaluate the binding free energies of the considered ubiquitin-complex systems, we performed Molecular Mechanics-Poisson–Boltzmann/Surface Area (MM-PBSA) calculations. These calculations were performed using pyBindE,^61,62^ which is a Python-based software specifically designed for conducting MM-PBSA calculations with GROMOS54A7 force field parameters.

### Knot Detection

The topological state of UCH-L1 was evaluated using the Kymoknot software tool,^63^ which can determine the topological state of a protein conformation and identify the knot type. For each frame of the MD trajectory, Kymoknot provides the knot presence confirmation and the number of residues on the threaded N- and C-terminus.

### Recombinant Protein Expression and Purification

The *wt* human UCH-L1 was cloned into a pET23a expression vector with a C-terminal His-tag, which was used as a template for generating two N-terminal truncated variants of UCH-L1 generated by site-directed mutagenesis as described previously.^30^ These constructs lacked two and five amino acid residues from their N-terminus named ΔN2 and ΔN5, respectively. DNA sequencing confirmed all the plasmid constructs (Mingxin Biotechnology, Taipei, Taiwan). For the ΔN2, the starting amino acid residue leucine was replaced with methionine for bacterial expression (ΔN2-L3M system). All the plasmid constructs were individually transformed into *E. coli* BL21 (DE3) strain (ECOSTM, 21, Yeastern Biotech Co., Taipei, Taiwan) for protein overexpression. The proteins were purified as described previously.^64^ Briefly, the transformed cells were induced with 0.5 mM IPTG at an OD_600_ of 0.6–0.8 and incubated at 16 °C for 18–20 h. The cell pellets were harvested by centrifugation and lysed by a Nanolyzer N2 (NanoLyzer) at 18 kpsi, and cell debris was removed by centrifugation. The target recombinant proteins were purified by Ni-NTA (Roche) chromatography followed by size-exclusion chromatography using HiLoad 16/600 Superdex 75 column (Cytiva, USA). The proteins were stored in a buffer containing 10 mM sodium phosphate (pH 7.4), 137 mM NaCl, 2.7 mM KCl, 1 mM TCEP, and 0.5 mM EDTA.

### Ubiquitin-AMC Assay

The deubiquitinase activity assay was conducted in a 384-well microplate at 30 °C. The proteins *wt*, ΔN2, and ΔN5 deubiquitination activities were analyzed using ubiquitin-7-amido-4-methylcoumarin (Ub-AMC) (Cat. # M3030; UBPBio, U.S.A.) as the substrate. Enzymes were mixed with a reaction buffer (50 mM Tris-HCl pH 7.6, 0.5 mM EDTA, 5 mM DTT, and 0.05% (v/v) BSA) containing varying Ub-AMC concentrations (0–300 nM). The final enzyme concentration was set to 2 nM in the reaction mix. Fluorescence was measured at an excitation wavelength of 380 nm and an emission wavelength of 460 nm in a 96-well microplate format using a plate reader (Infinite M1000 pro, Tecan, Switzerland). The fluorescence measurements were determined every 5 s for 1000 s. The initial rates of the reaction of the proteins were plotted against substrate concentration and fitted to the Michaelis–Menten equation using Prism v10.0 (GraphPad, U.S.A.).

### Circular Dichroism (CD) Spectroscopy

The far-UV CD spectra of *wt* UCH-L1 and its variants (ΔN2 and ΔN5) were collected at 25 °C in a CD spectrometer (J-815, Jasco, Japan). The protein concentration was set to 6 μM in a buffer containing 10 mM sodium phosphate (pH 7.4), 137 mM NaCl, 2.7 mM KCl, 1 mM TCEP, and 0.5 mM EDTA. Each spectrum was collected with three accumulated scans spanning between 195 and 260 nm with an interval of 0.5 nm in a quartz cuvette of 1 mm path length (Helma, Germany). The built-in multivariate analysis program of JASCO Spectra Manager was used to estimate the secondary structure content of the proteins.

### Nanodifferential Scanning Fluorimetry (nanoDSF)

Thermal melting analysis of *wt* UCH-L1, ΔN2, and ΔN5 was conducted using a Tycho NT.6 instrument (NanoTemper Technologies). All samples had a protein concentration of 8 μM and were prepared in a buffer containing 10 mM sodium phosphate (pH 7.4), 137 mM NaCl, 2.7 mM KCl, 1 mM TCEP, and 0.5 mM EDTA. The samples were heated in glass capillaries with a linear thermal ramp of 30 °C/min, ranging from 35 to 95 °C. Tryptophan fluorescence at 330 and 350 nm was recorded during heating. Data analysis and derivative calculations were performed using the NT.6 instrument’s automated evaluation features.

## Results and Discussion

In the present study, we aim to explore the dynamic behavior of UCH-L1 and investigate the role of the 5_2_ knot in the structural stability of the protein’s catalytic site. To do so, we started by performing an MD simulation study of the apo and holo (ubiquitin complex) forms of the protein, as well as of the several N- and C-terminal truncated variants of UCH-L1, and single-point mutants.

The structural characterization of both the apo and holo forms during the MD simulations was performed by using several metrics such as the RMSD, radius of gyration, secondary structural content, and the number of threaded residues (Figures S1–S4 of the Supporting Information). An evaluation of the interfacial area and the MM-PBSA binding energy of the complex was also performed (Figures S5–S6 of the Supporting Information). Structural properties were found to exhibit low deviations and displayed relatively rapid convergence, attaining a stable plateau within the initial 100 and 200 ns for the apo and holo systems, respectively (Figures S1–S3 of the Supporting Information). However, the number of threaded residues required a longer convergence time, which is especially evident in the holo *wt* system (see Figure S4 of Supporting Information). Thus, to ensure robust equilibration, we adopted a conservative approach and discarded the initial 250 and 500 ns for the apo and holo systems, respectively.

### Size of Threaded Termini in Dynamic Conformations of UCH-L1

**Figure 3:**
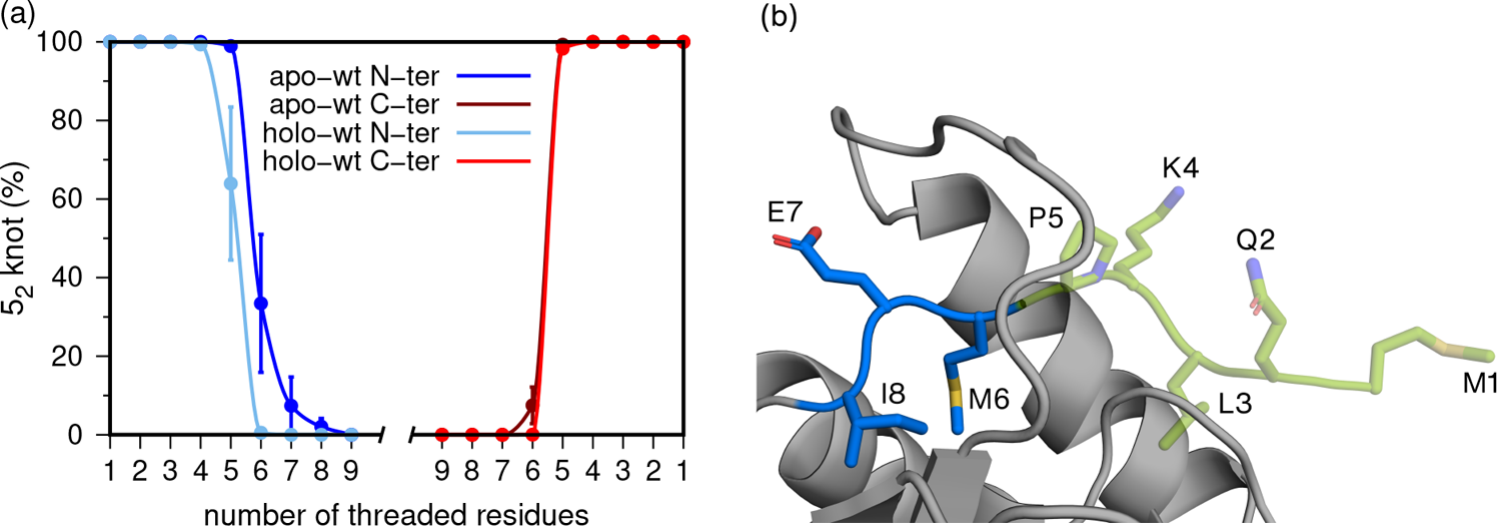
Frequency of 5_2_ knotted conformations (in the apo and ubiquitin-UCH-L1 complex) with 1 to 9 threaded residues (a) and graphical representation of the N-terminal residues (b). In the case of the C-terminus, residue 223 was renumbered as residue 1, and residue 215 was renumbered as residue 9. In the cartoon/sticks representation, the knotted core is shown in gray, and the N-terminus is colored in blue and green (threaded part).

As previously mentioned, the application of knot detection methods to the crystal structure of protein UCH-L1 indicates that it embeds a shallow 5_2_ knot, being necessary to remove 5 and 6 residues from the C- or N-terminus, respectively, to unknot the chain. Since a single threading movement of one of the termini is required to remove the 5_2_ knot, we started by exploring the stability of the native knotted topological state, in the apo and holo states, by investigating the dynamic behavior of the threaded termini along the MD trajectories. In particular, using the Kymoknot software tool,^63^ we quantified the number of threaded residues at both ends. For both the apo and holo states, we calculated the frequency for the number of threaded residues (from 1 to 9), in the ensemble of conformations identified as being knotted (Figure 3a). In the apo state, all sampled conformations exhibit threaded termini with 5 residues (Figure 3b). However, in a significant fraction of the apo conformations (≈ 30%), there are 6 threaded residues at the N-terminus, and it is yet possible to find 7 threaded residues at the N-terminus in about 10% of the sampled conformations. In the timescale assessed with the MD simulations, the movement of terminal residues appears to be considerably more restricted at the C-terminus; indeed, in this case, and contrary to the starting crystal structure, one could not find conformations with 6 (or more) threaded residues. In the presence of ubiquitin (i.e., in the holo state), the dynamics of the C-terminal residues remain mostly unchanged, but significant differences are observed at the N-terminus. All sampled conformations exhibit 4 threaded residues at the N-terminus, and no conformation was found with 6 threaded residues. This phenomenon is likely induced by the binding of ubiquitin in the protein’s catalytic pocket, causing a conformational rearrangement immediately adjacent to the N-terminal region.

### Truncated Termini and Knot Stability

**Figure 4:**
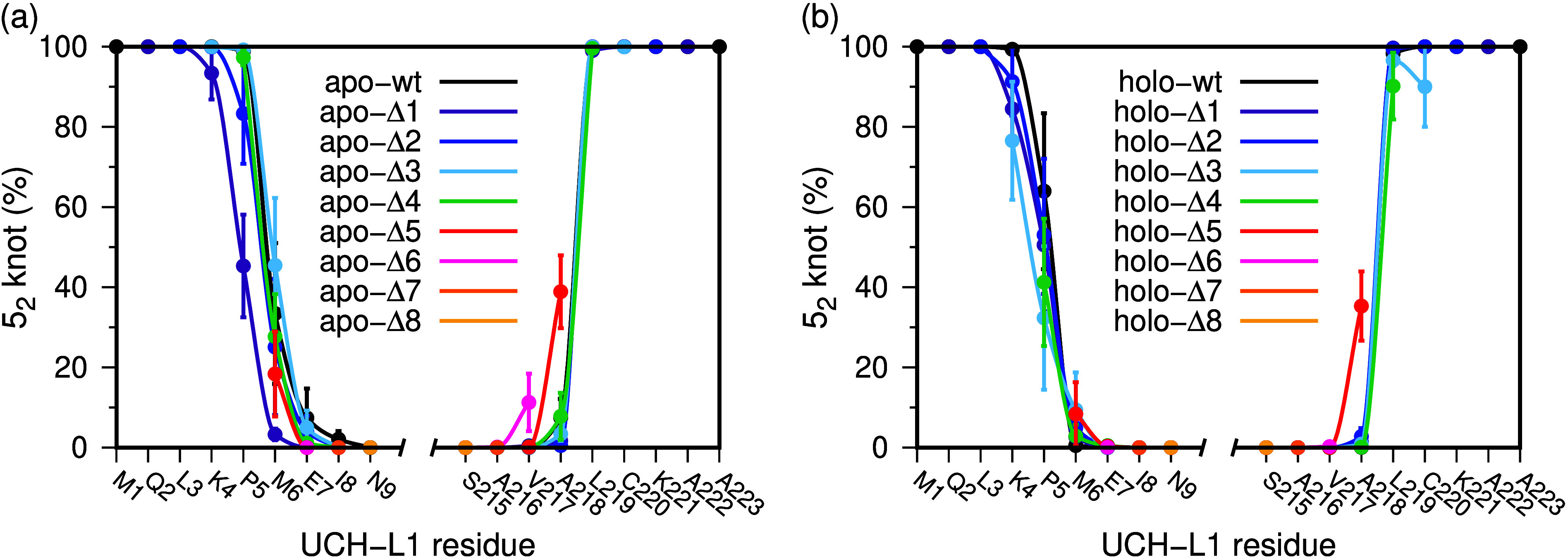
Probability to find 5_2_ knotted conformations with a specific threaded residue in N- and C-terminal truncated variants of the apo (a) and holo (b) forms of UCH-L1. The apo- and holo-*Δi* correspond to truncated variants in the apo or ubiquitin-complex state with 1≤ *i* ≤ 8 residues removed from the N- or from the C-terminus. Note that the considered threaded residue depends on the truncated variant. For example, in the case of apo-Δ1, which lacks the first residue, the knotting probability profile only starts on residue 2 (or 222 for the variant truncated at the C-terminus).

The motivation behind performing a set of deletions at both the N- and C-terminus was to render these regions more labile, thereby facilitating unthreading events that may disrupt the knotted topological state in a timescale accessible via MD. We observe that the removal of up to 4 residues (i.e., the stretch M(1)Q(2)L(3)K(4)) from the N-terminus does not affect the topological state, with the truncated variant apo-Δ4 preserving the 5_2_ knot in 100% of the sampled conformations in the considered timescale. In the vast majority of these conformations, the first threaded residue is P5, and in 30% of them, there are two threaded residues, namely, P5 and M6. This observation indicates that the removal of the first four N-terminal residues only slightly increased the mobility of the N-terminus and that its movement occurred outward, i.e., in the opposite direction of the protein’s core (Figure 4a). By removing the first five N-terminal residues the protein’s crystal structure becomes unknotted according to Kymoknot. We still find a small fraction (about 20%) of knotted conformations with the 6^*th*^ residue (M6) threaded, against the 30% found in the *wt*, but these may just be a direct consequence of starting all simulations from the knotted conformation. In the sub-μs timescale of the MD simulations, the N-terminal region is remarkably stable. However, we should not put aside the possibility the simulated structures may not represent accurate descriptions of those observed experimentally, which form in timescales not accessible to MD simulations.

Similar results are obtained when UCH-L1 forms a complex with ubiquitin in the holo state (Figure 4b), as well as for the truncated variants at the C-terminus. The C-terminal region is even more structurally stable than the N-terminal region, with only two truncated variants (Δ5 and Δ6) exhibiting a non-negligible probability of being knotted as a result of threading movements of the truncated C-terminal part.

### The Role of the 5_2_ Knot on the Structural Stability of the Catalytic Site

#### Computational Results

**Figure 5:**
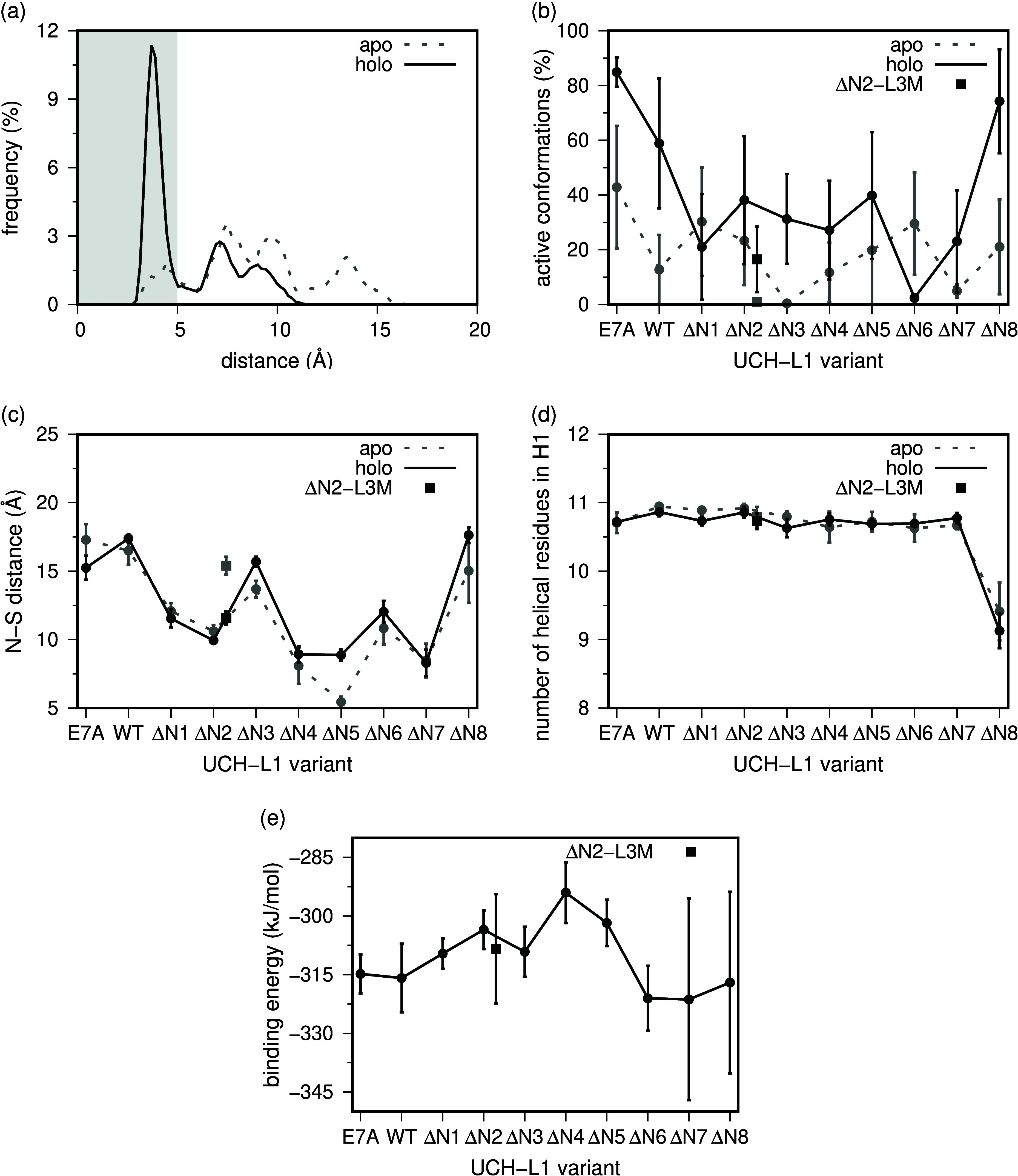
Distance histogram between Cys90 and His161 for the *wt* system (a), the percentage of active conformations (previous distance <5 Å) involving each of the mutant and truncated variants of UCH-L1 (b), the distance between the N-terminus and Cys90 (N–S distance) (c), the number of helical residues present in helix-1 (d), and the MM/PBSA binding energy between UCH-L1 and ubiquitin (e).

The N-terminus is the most proximal to the catalytic pocket of UCH-L1 (∼15 Å vs ∼30 Å for the C-terminus). Furthermore, as indicated by the data in the preceding section, there is (on average) one less N-terminal residue threaded in the holo-form (Figure 3). These findings suggest that the optimal catalytic activity of UCH-L1 may depend on a conformational rearrangement of the N-terminus. Hence, we looked at the role played by the N-terminus, and by the 5_2_ knot, in the structural stability of UCH-L1’s catalytic site. More precisely, we focused our analysis on the structural stability of UCH-L1’s catalytic triad. The latter consists of Cys90, His161, and Asp176, with the imidazole group of the histidine acting as a general base and enhancing the cysteine nucleophilicity. To activate UCH-L1, these two groups need to come closer (<5 Å). The comparison of the crystal structures of the holo- and apo- forms (Figure 2) suggests that the approximation of the functional groups may be driven by ubiquitin-binding, and the results from the MD simulations corroborate this hypothesis. Indeed, the frequency distribution for the C90–H161 distance (calculated between the sulfur atom and the closest imidazole nitrogen atom) is significantly different between the apo- and holo-systems (Figure 5a), with the former having a clear peak at (functional) distance ∼4 Å, and the latter being only able to scarcely populate these active conformations. A cutoff distance of 5 Å provides a quantification of the percentage of active conformations in the *wt*, the E7A mutation, and in the truncated versions of UCH-L1 (Figure 5b and Figure S7 of the Supporting Information). In the *wt*, a clear activation of the protease is observed upon substrate binding (∼20% in *wt* vs ∼60% of the holo form). A similar trend was observed for the E7A mutant, indicating that the reported decrease in deubiquitinase activity^41^ is not induced by an active site misalignment. However, this substrate-induced activation is significantly reduced when just a couple of residues are truncated from the protein’s N-terminus (e.g., ΔN2-L3M). Considering that the MD simulations confirm the presence of the 5_2_ knot in all ΔN1 to ΔN4 truncated versions, these results thus indicate that the integrity of the N-terminus is an important factor for the activation of UCH-L1.

We noticed an unexpected increase in the population of active conformations for ΔN8. The latter is likely driven by some structural rearrangement resulting from the loss of helical content at the N-terminal region, similar to what has been observed for ΔN11.^30^ This effect is well illustrated by following the distance between the N-terminus and the catalytic C90 (Figure 5c), where the unexpected increase observed for ΔN8 can be rationalized by the partial unfolding of this terminal region. Indeed, the DSSP tool confirmed the loss of two helical residues in the N-side of helix-1 of the ΔN8 truncated version (Figure 5d). The MM/PBSA results indicate a slight increase in binding energy from ΔN1 to ΔN4, followed by a decrease from ΔN5 to ΔN8 (Figure 5e). Notably, this recovery of the binding affinity seems to be correlated with the loss of the knotted structure (Figure 4). The high error bars in ΔN7 and ΔN8 are justified by the presence of abnormally high variability between replicates, beyond what is commonly observed (Figure S6 of the Supporting Information), and with different levels of unfolding in helix-1, for ΔN8, which alters the local region around the catalytic cleft and the binding. The unperturbed binding affinity of the E7A system does not correlate with the high importance of the Glu residue for ubiquitin binding to UCH-L1 found experimentally^41^ and suggests that the instability introduced by the E7A mutation goes beyond an electrostatic effect, which is what is captured in our MM-PBSA protocol.

#### *In Vitro* Results

To validate our computational predictions, we conducted *in vitro* analyses. As previously stated, we restricted the experimental investigations to ΔN2 and ΔN5. We note that in the case of ΔN5, the interpretation of the computational results is strongly limited by the timescale covered by the MD simulations, and by complementing the latter with experiments, one expects to get a better understanding of the impact of truncating the five N-terminal residues on structure and function. We thus expressed the *wt* protein, together with the ΔN2-L3M and ΔN5 truncated variants by site-directed mutagenesis.

**Figure 6:**
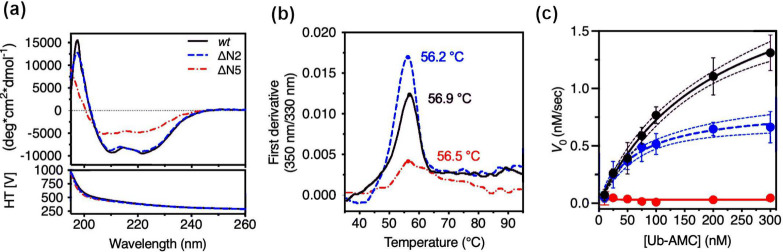
Biophysical and biochemical assessments of the impacts of N-terminal truncation on the structure and function of UCHL1. (a) Far-UV CD spectra of UCH-L1 wt, ΔN2, and ΔN5. (b) Inflection temperature (T_i_ °C) of UCH-L1 wt, ΔN2, and ΔN5 as a function of increasing temperature. The peak in the first derivative ratio (350 nm/330 nm) corresponds to the detected T_i_ value of the sample. (c) Michaelis–Menten analysis of Ub-AMC hydrolysis catalyzed by UCH-L1 *wt*, ΔN2, and ΔN5. The experiments were carried out in five technical replicates, and the mean values and the standard deviations are shown with the 90% confidence intervals shown in dashed lines. The data points of *wt*, ΔN2, and ΔN5 are colored black, blue, and red, respectively.

Comparison of the far-UV CD spectra of the UCH-L1 *wt*, ΔN2, and ΔN5 revealed differences for ΔN5 with respect to *wt* and ΔN2 (Figure 6a). Quantitative estimation of the secondary structure contents based on the CD spectra indicated a significant loss of α-helix and increase in β-sheet for ΔN5 whereas no appreciable changes were observed for ΔN2 (Table 1).

**Table 1:** Secondary Structure, Thermal Melting, and Michaelis–Menten’s Parameters of UCH-L1 *wt*, ΔN2, and ΔN5

Protein	α-helix (%)	β-sheet (%)	T_i_ (°C)	K_M_ (nM)	*k*_cat_ (s^–1^)	*k*_cat_/K_M_ (s^–1^ M^–1^)	
*wt*	28.0	24.9	56.9	211 ± 37	2.25 ± 0.22	1.1 ± 0.1 × 10^8^	
ΔN2	26.7	26.8	56.2	63 ± 15	0.84 ± 0.08	1.3 ± 0.2 × 10^8^	
ΔN5	13.1	33.4	56.5	–	–	–	

The destabilization of the ΔN5 system is not replicated by the computational data (Figure 5d and Figure S8 of the Supporting Information), which indicates that the secondary structure is conserved within the microsecond timescale despite the low percentage of conformations exhibiting the 5_2_ knot. These results highlight the limitation of MD simulations in replicating the conformational space associated with differences in secondary structure content. In the particular case of the simulations carried out in this work, this limitation is rooted in two major aspects: (1) the structural bias toward the native structure resulting from building all truncated variants from the *wt* X-ray structure; and (2) the timescale spanned by the MD simulations (microseconds), which is not enough to detect large scale conformational movements such as those associated with changes in secondary structure content.

Within the covered timescale, we observed helicity loss for ΔN8. To search for other trends in the computational data that could hint at the experimental observations, we also analyzed the RMSF values per residue in the ΔN5 and ΔN8 truncated variants, and compared it to *wt* and ΔN2-L3M (Figure S9 of the Supporting Information). However, all systems showed an RMSF profile similar to *wt*, with only ΔN8 exhibiting increased fluctuations in specific regions: the helix-1, which lost most of its N-flanking segment; the loop connecting to Cys90 (residues 81–90), which is usually stabilized by the truncated N-terminus segment; and helix-4 (residues 111–120) that is well exposed to the solvent.

We further investigated the impact of N-terminal truncation of ΔN2, and ΔN5 on the deubiquitinase activity of UCH-L1 using the Ub-AMC assay.^65^ Michaelis–Menten kinetics analyses of UCH-L1 variants showed that ΔN2 exhibited a lower K_M_ (∼63 nM) compared to that of *wt* (211 nM) whereas the *k*_cat_ of ΔN2 was reduced by 2.5-fold compared to *wt*, indicating that the minimal N-terminal truncation can significantly affect the enzymatic activity of UCHL-1 (Figure 6b) and Table 1). In the case of ΔN5, where the protein most likely tends to unknot, the functional activity was abolished entirely, rendering it nonfunctional. The mismatch between these results and the MM-PBSA calculations (Figure 5e) exposed again some of the limitations of this computational approach already reported.^66^ In particular, the MM-PBSA method will not provide a good estimate of the binding energy when a large conformational transition occurs and, due to timescale limitations, is not well captured by the MD sampling. When these conformational transitions are partially captured in some replicates, this leads to larger error bars like those obtained for ΔN7 and ΔN8.

Overall, our findings emphasize the significance of the correct conformation adopted by the N-terminus and the presence of the 5_2_ knot in the native structure, since both factors appear to contribute in different ways (catalytic triad alignment and ubiquitin-binding), and may work in tandem to ensure proper catalytic activity.

## Conclusions

During the last two decades, researchers in protein science have dedicated a significant effort toward understanding the role played by knotted topologies in protein structure and function, and determining whether knots may provide some functional advantage to their carriers.^1,10,12^ The picture emerging from these studies is not necessarily clear, with simulations and experiments often providing contradicting results (e.g., in what regards thermal, mechanical, and kinetic stability).^12^ In the particular case of enzymes, it has been suggested that knots contribute to shape their active sites,^67−69^ or even alter enzymatic activity.^34^ Interestingly, in the particular case of the SPOUT class of methyltransferases, simulation, and experimental data agree the knotted structure adopts a unique way of binding ligands.^70,71^

A major experimental challenge in investigating the role of protein knots is constructing control systems that make it possible to directly access the effects of topology on folding properties. An adequate control system should preserve (to the largest possible extent) the geometry of the native structure. While this may be trivially achieved in computational models (e.g., in lattice systems by performing some change on the connectivity of the chain^14^), it represents a major experimental challenge.^72,73^

The major goal of the present work was to explore the role of the 5_2_ knot in the structural stability of the catalytic triad of protein UCH-L1 and, consequently, on its enzymatic activity. To do so, we originally deployed Molecular Dynamics (MD) simulations of wild-type UCH-L1, and several truncated variants lacking (from 1 to 8) N-terminal residues. The MD simulations show that, in the microsecond timescale, the movement of terminal residues appears to be considerably more restricted at the C-terminus, even when the N-terminus is truncated. This is in good agreement with the observation that the C-terminal part of UCH-L1 is, in general, more stable than the N-terminal region and that the mechanical pulling from different directions does make a difference in the translocation of UCH-L1 through the ClpXP pore.^45^

More interestingly, the MD simulations also reveal the somehow unexpected result that the removal of the first two (or even just one) N-terminal residues has dramatic consequences on the population of catalytically active conformations (probed indirectly in the simulations by measuring the distance between residues C90–H161) while preserving the secondary structural content, and topological state of the native structure. The experimental results based on a Michaelis–Menten analysis do confirm this theoretical prediction with the k_*cat*_ of the truncated mutant lacking two residues at the N-terminus showing a 2.5-fold reduction relative to the *wt* UCH-L1. Additionally, the MD simulations indicate that the truncation of five N-terminal residues substantially increases the probability of unknotting events, with the vast majority of sampled conformations being unknotted. The experimental results further show that this truncated variant exhibits significant changes at the level of the secondary structural content, becoming nonfunctional.

Overall, we observed that the integrity of the N-terminus is crucial for the catalytic triad alignment and ubiquitin binding. However, a direct correlation between the knotted topology and UCH-L1 activity is difficult to establish. Considering the possibility that the truncated variant folds into a topologically knotted conformation, the loss of activity could be directly associated with the observed change in secondary structural content. The alternative scenario, which we find more likely based on the relatively small fraction of knotted conformations found for ΔN5 in the simulations, is that this truncated variant is not able to fold into the knotted topological state. In this case, we can interpret our results by considering that the knotted topological state contributes to maintaining the secondary structural content of the protein, therefore playing a role in the structural stabilization of the native structure. In line with this reasoning, we can further hypothesize that the presence of the knot in the native structure contributed to the evolution of the N-terminal sequence’s composition, in the sense that this enzyme has evolved its N-terminus structure toward ubiquitin-binding efficiency and active site alignment, rather than stability in its secondary structure, which can be at least partially provided by its knotted topology.

## Data Availability

The GROMACS package is freely available software used to perform MD simulations and can be downloaded at https://manual.gromacs.org/documentation/2020.1/download.html. PyMOL v2.5 is also free software for molecular visualization and generating high-quality images. It can be downloaded from https://pymol.org/2. A zip file with all topologies, system configurations, and parameter files is also provided in the Supporting Information.

## Supporting Information Available

Time series for RMSD, radius of gyration, secondary structure, number of threaded residues, complex interface contact area, and MM/PBSA binding energies. Distance histogram between Cys90 and His161 for all systems (PDF)
All topologies, system configurations, and parameter files (ZIP)
