# Supplementary material for: Is There a Functional Role for the Knotted Topology in Protein UCH-L1?
Source: J Chem Inf Model. 2024 Jul 24;64(17):6827–37. doi: 10.1021/acs.jcim.4c00880 (PMC11388461; doi:10.1021/acs.jcim.4c00880)
Supplement: Supplementary file 1 — ci4c00880_si_001.pdf [file ci4c00880_si_001.pdf]

Supporting Information: Is there a functional role for the knotted topology in protein UCH-L1?

Sara G. F. Ferreira,[†] Manoj K. Sriramoju,[‡] Shang-Te Danny Hsu,[‡,¶,‖] Patrícia F. N. Faísca,[*,§] and Miguel Machuqueiro[*,†]

†BioISI – Instituto de Biossistemas e Ciências Integrativas, Departamento de Química e Bioquímica, Faculdade de Ciências, Universidade de Lisboa, 1749-016, Lisboa, Portugal

‡Institute of Biological Chemistry, Academia Sinica, Taipei, 11529, Taiwan

¶International Institute for Sustainability with Knotted Chiral Meta Matter (WPI-SKCM^2), Hiroshima University, 1-3-1 Kagamiyama, Higashi-Hiroshima, Hiroshima 739-8526, Japan

§BioISI – Instituto de Biossistemas e Ciências Integrativas, Departamento de Física, Faculdade de Ciências, Universidade de Lisboa, 1749-016, Lisboa, Portugal

‖Institute of Biochemical Sciences, National Taiwan University, Taipei, 11529, Taiwan

E-mail: pffaisca@ciencias.ulisboa.pt; machuque@ciencias.ulisboa.pt

Phone: +351-21-7500112

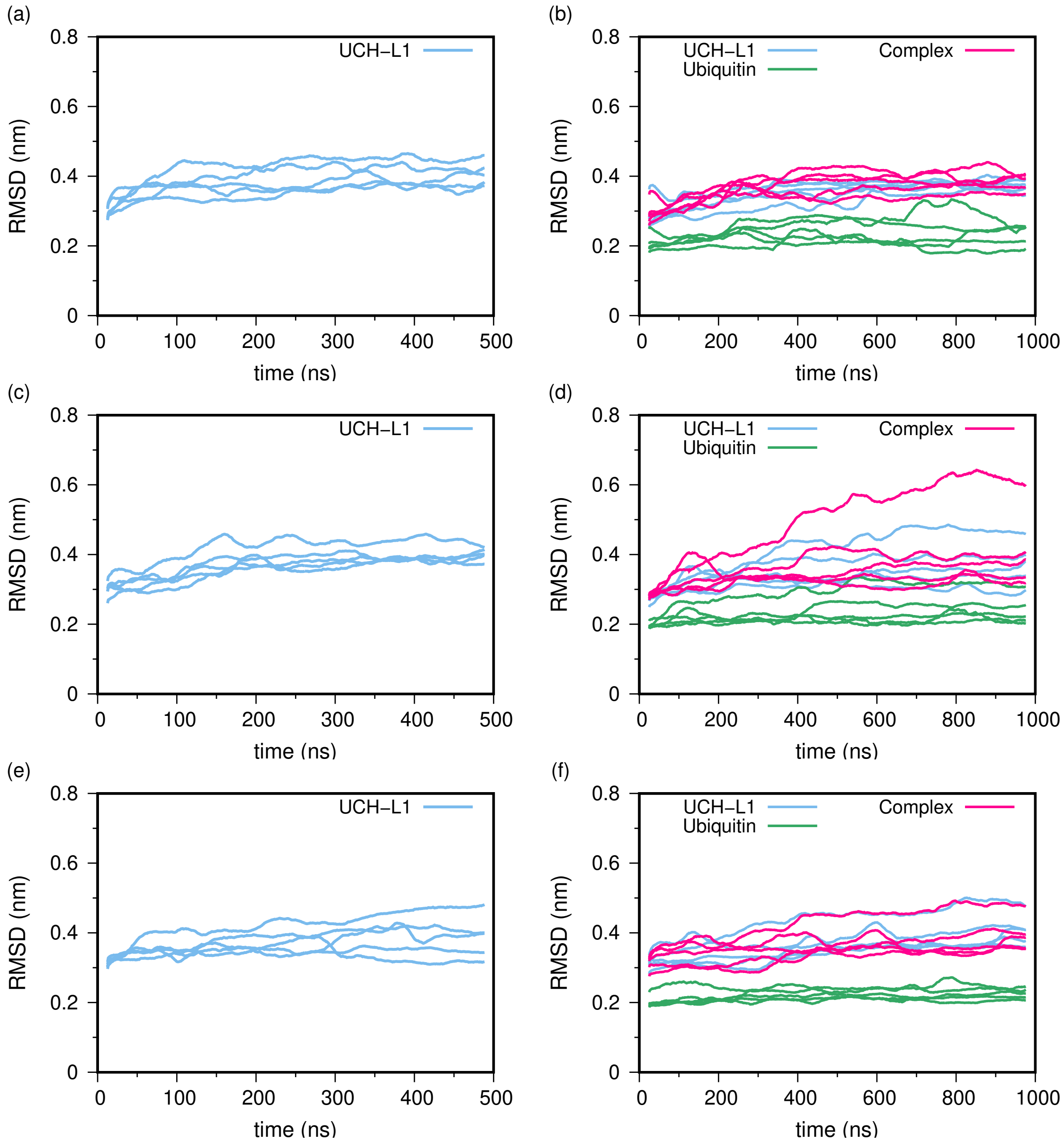

Figure S1: Root Mean Square Deviation of the apo (a, c, e) and holo (b, d, f) states for *wt* (a,b) ΔN2 (c, d), and ΔN5 (e, f) systems. UCH-L1 is represented in blue, Ubiquitin is represented in green, and the complex (UCH-L1 + Ubiquitin) is represented in pink. The five replicates are shown. Data was averaged using a floating window of 25 ns and 50 ns for the apo and the holo systems, respectively.

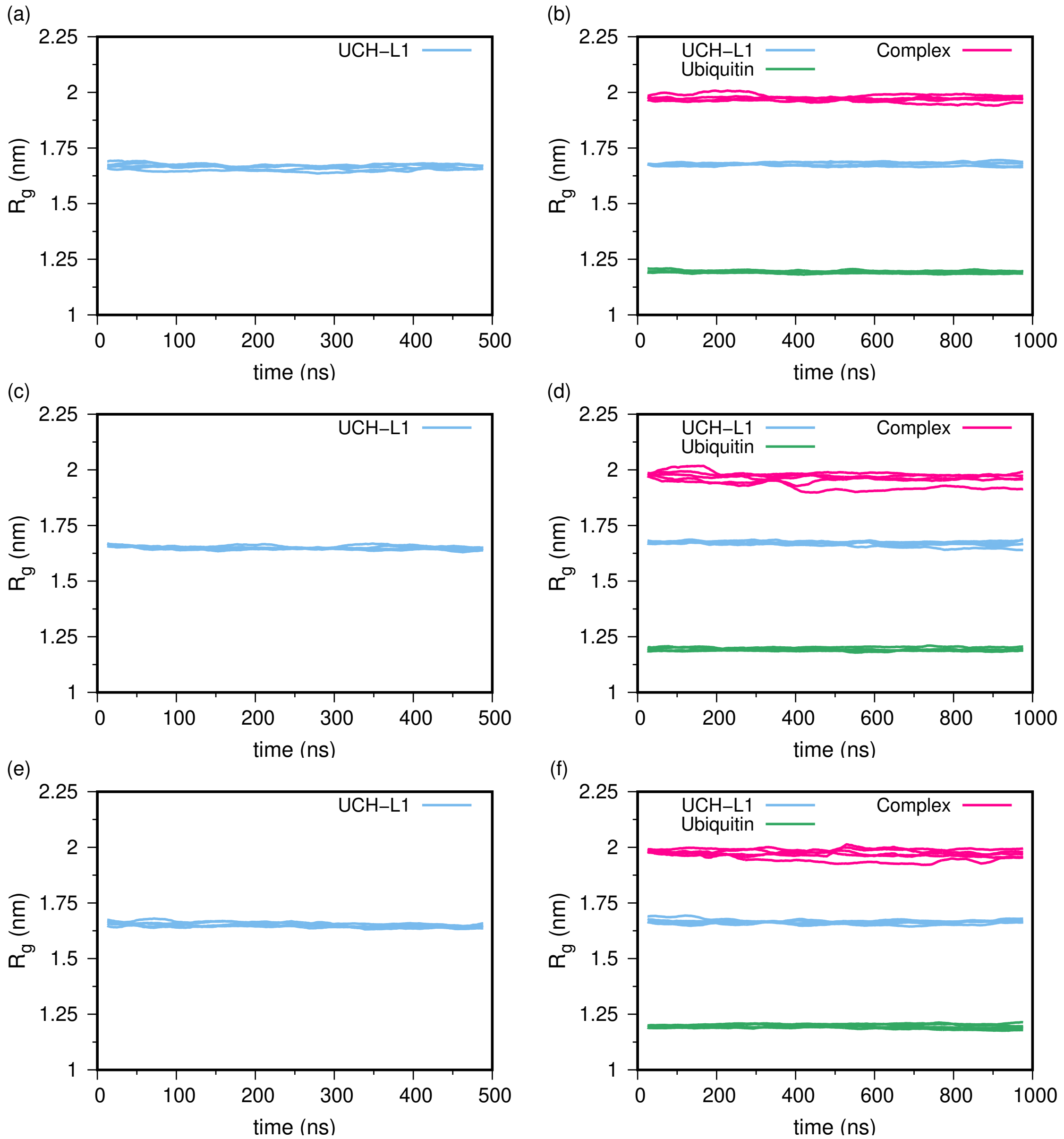

Figure S2: Radius of gyration of the apo (a, c, e) and holo (b, d, f) states for *wt* (a,b) ΔN2 (c, d), and ΔN5 (e, f) systems. UCH-L1 is represented in blue, Ubiquitin is represented in green, and the complex (UCH-L1 + Ubiquitin) is represented in pink. The five replicates are shown. Data was averaged using a floating window of 25 ns and 50 ns for the apo and the holo systems, respectively.

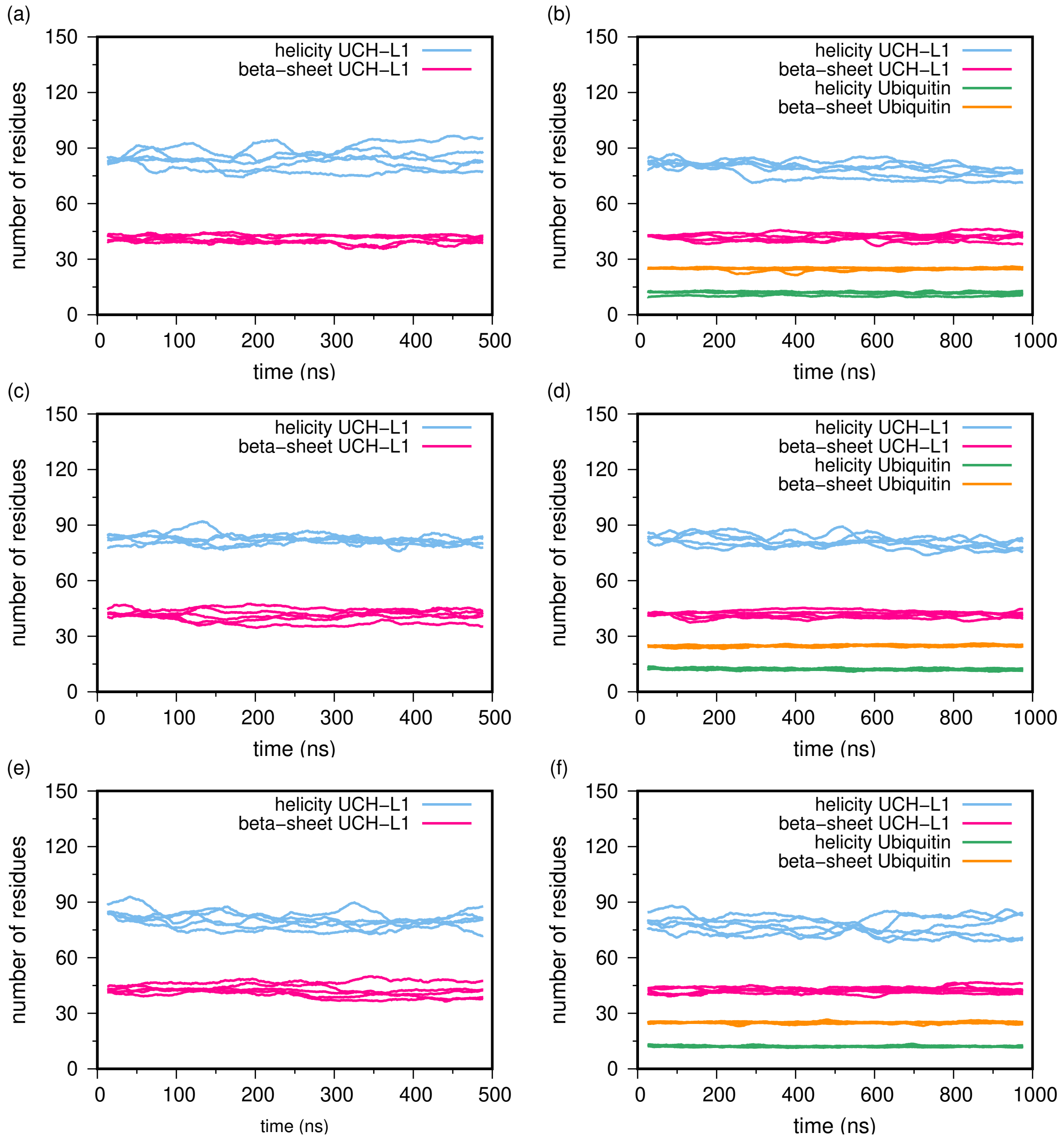

Figure S3: Secundary structure, using the DSSP criteria, of the apo (a, c, e) and holo (b, d, f) states for *wt* (a,b) ΔN2 (c, d), and ΔN5 (e, f) systems. α-helices are represented in blue and green, regarding UCH-L1 and Ubiquitin, respectively, and β-strands are represented in pink and orange, regarding UCH-L1 and Ubiquitin, respectively. The five replicates are shown. Data was averaged using a floating window of 25 ns and 50 ns for the apo and the holo systems, respectively.

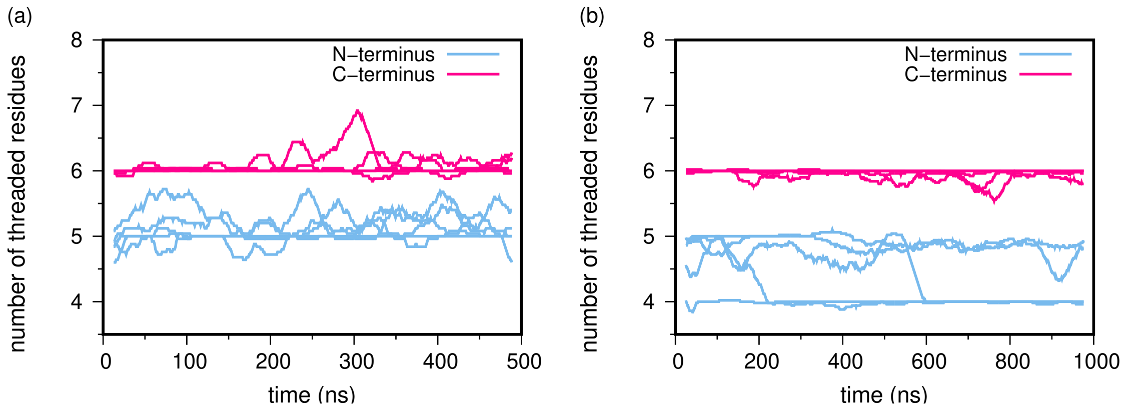

Figure S4: Number of threaded residues in the apo (a) holo (b) systems. The number of residues involved in the N-terminus is represented in blue, while pink represents the C-terminus. The five replicates are shown. Data was averaged using a floating window of 25 ns and 50 ns for the apo and the holo systems, respectively.

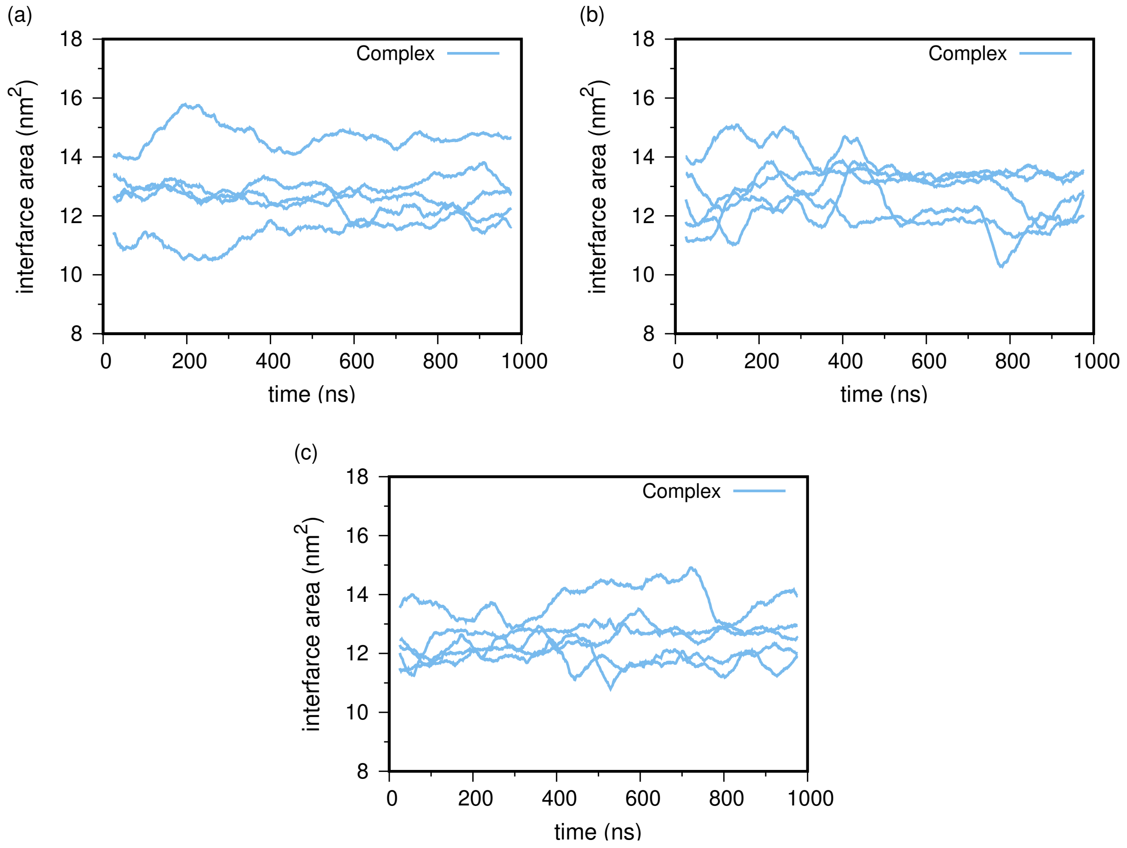

Figure S5: Contact interface area between UCH-L1 and ubiquitin in *wt* (a), ΔN2 (b), and ΔN5 (c) systems. The five replicates are shown.

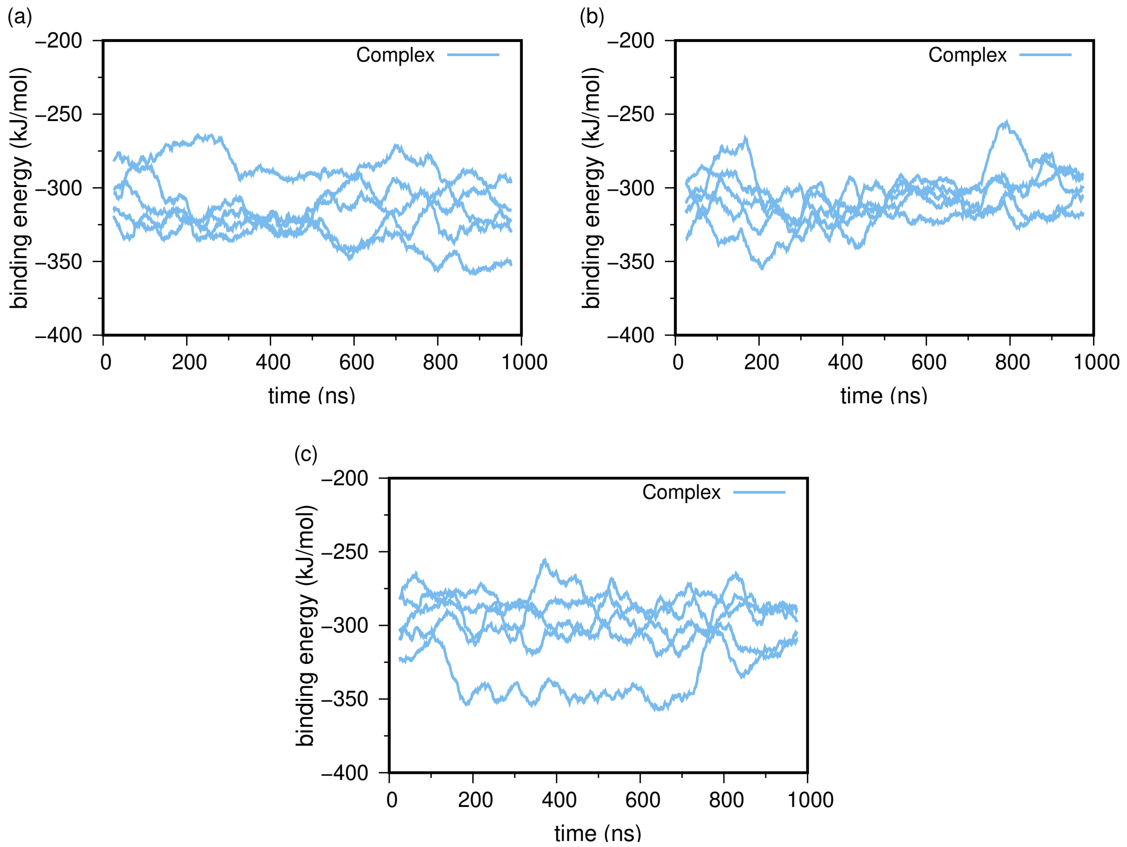

Figure S6: Binding Free Energy between UCH-L1 and ubiquitin in the *wt* (a), ΔN2 (b), and ΔN5 (c) systems. The five replicates are shown.

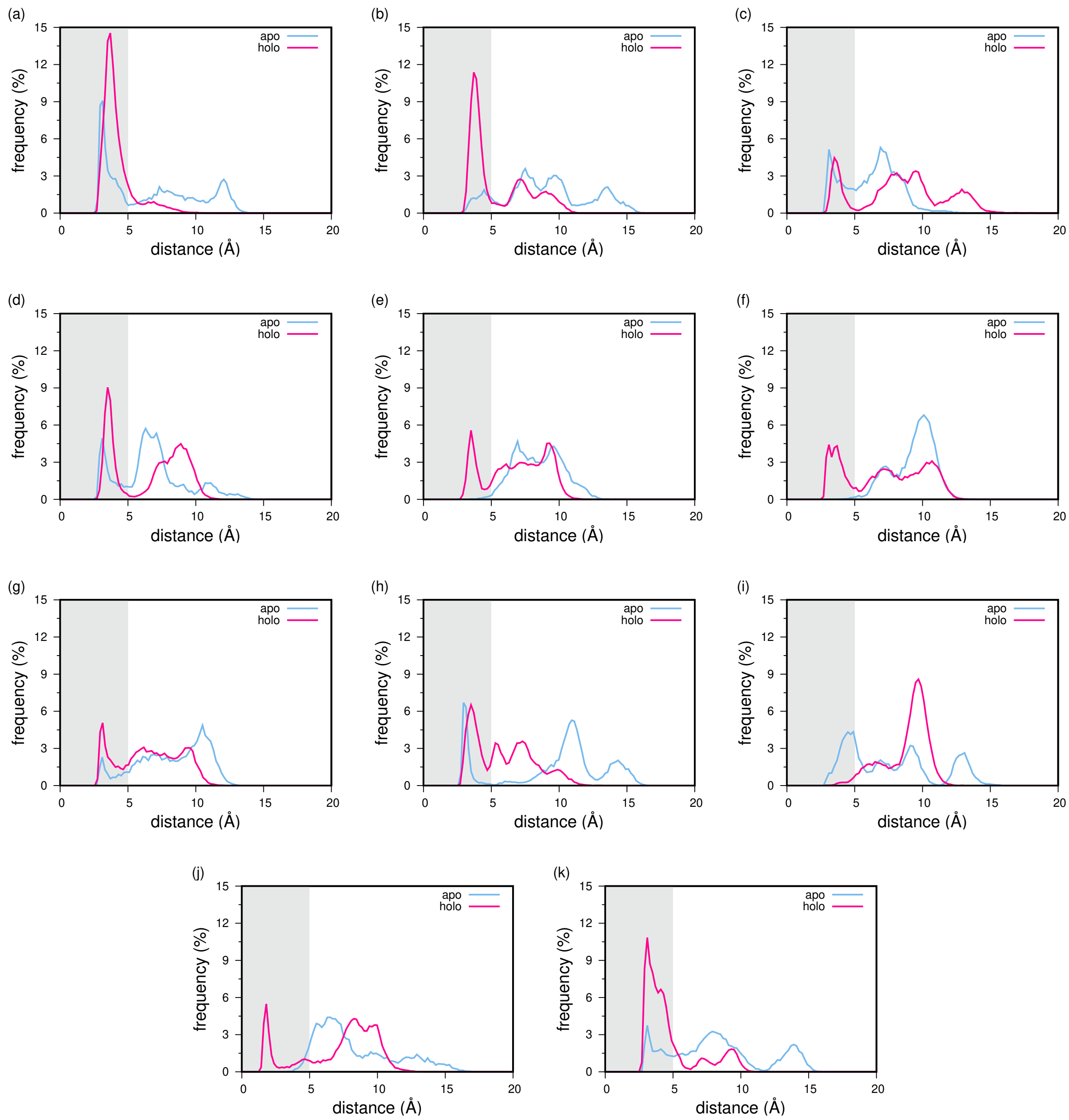

Figure S7: Distance histogram between Cys90 and His161 for E7A (a), *wt* (b), ΔN1 (c), ΔN2 (d), ΔN2-L3M (e), ΔN3 (f), ΔN4 (g), ΔN5 (h), ΔN6 (i), ΔN7 (j), ΔN8 (k) systems.

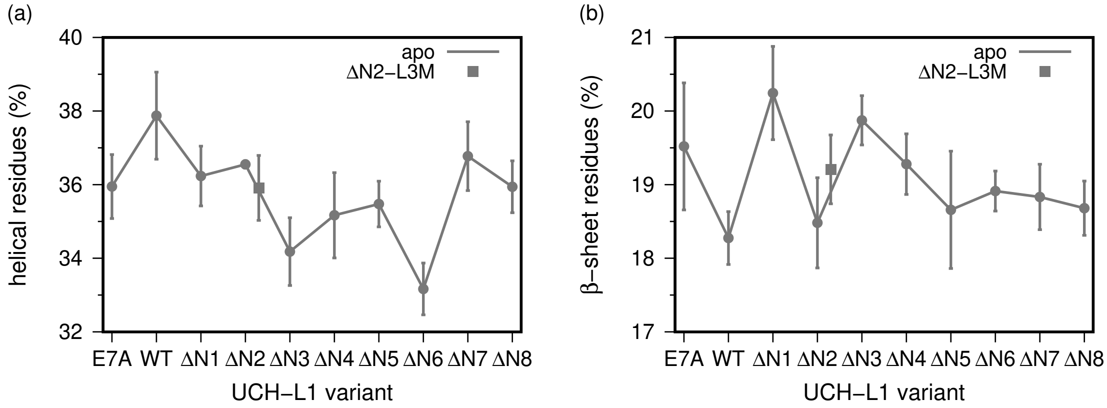

Figure S8: Percentage of helical (a) and β-sheet (b) residues present in the apo state of UCH-L1 involving the *wt*, mutants, and truncated variants of UCH-L1.

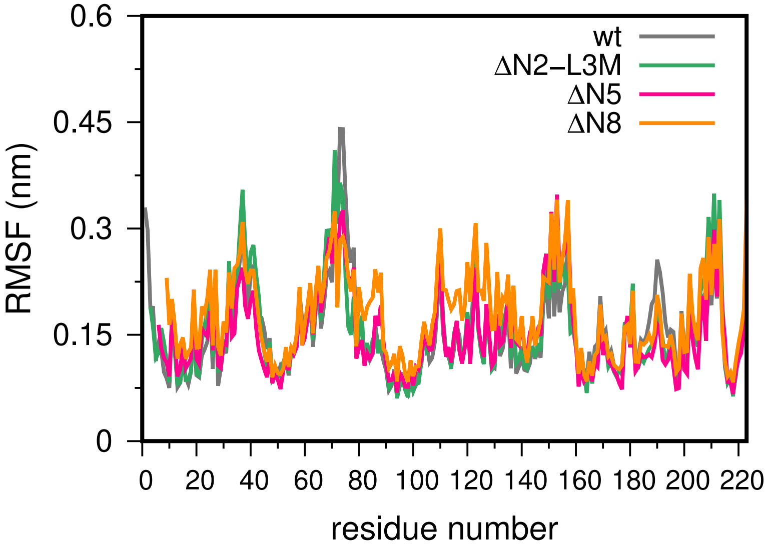

Figure S9: Root Mean Square Fluctuation per residue of the apo state of UCH-L1 *wt* (grey), ΔN2-L3M (green), ΔN5 (pink), and ΔN8 (orange).
